# A wearable perspiration biosensor for continuous biomarker monitoring in inflammatory bowel disease: Feasibility, concordance, and exploratory temporal dynamics

**DOI:** 10.1002/ctm2.70896

**Published:** 2026-11-14

**Authors:** Annapoorna Ramasubramanya, Kai‐Chun Lin, Sarah Shahub, Alexandra R. McDermott, Zachary D. Fine, Robert P. Hirten, Jessica Whang, Drew Helmus, Sriram Muthukumar, Bruce E. Sands, David T. Rubin, Shalini Prasad

**Affiliations:** ^1^ Bioengineering University of Texas at Dallas Richardson Texas USA; ^2^ The University of Chicago Medicine Inflammatory Bowel Disease Center Chicago Illinois USA; ^3^ The Dr. Henry D. Janowitz Division of Gastroenterology Icahn School of Medicine at Mount Sinai New York New York USA; ^4^ EnLiSense LLC Allen Texas USA

**Keywords:** biomarkers, Crohn's disease, inflammatory bowel disease, ulcerative colitis, ultradian rhythm, wearable devices

## Abstract

**Background:**

Serum C‐reactive protein (CRP) and faecal calprotectin, used to monitor inflammatory bowel disease (IBD) between endoscopies, require intermittent blood or stool sampling. We assessed whether a skin‐worn perspiration sensor could measure CRP and calprotectin continuously; TNFα and IL‐6 were exploratory analytes.

**Methods:**

At three US centres, perspiration biomarkers were recorded continuously for 3–30 days in 52 IBD patients (27 ulcerative colitis, 24 Crohn's disease, 1 indeterminate colitis) and 19–32 days in 9 controls, with serum CRP and faecal calprotectin sampled at baseline and study end. Agreement was assessed using correlation and Bland–Altman analysis; ROC‐derived cutoffs matched serum CRP 5 mg/L and faecal 250 µg/g. Circadian rhythmicity and ultradian (4–12 h) power were compared among biochemically active IBD (serum CRP > 5 mg/L; *n* = 15), inactive (*n* = 37), and controls.

**Results:**

47 of 52 IBD participants (90.4%) completed the protocol. Across 99 paired observations, perspiration CRP correlated with serum CRP (*r* = .52–.58); perspiration and faecal calprotectin correlated in ulcerative colitis (*r* = .59–.62) but not in Crohn's disease. A perspiration CRP cutoff of 1551 pg/mL (serum 5 mg/L) gave 85.7% sensitivity and 89.4% specificity; a calprotectin cutoff of 385 (µg/g)·min (faecal 250 µg/g) gave 92.3% and 67.9%. Negative predictive values were high (95%–97%), positive modest (57%). Circadian MESOR and amplitude of both markers were higher in active than inactive IBD. Ultradian power differed by group for calprotectin (Kruskal–Wallis *p* = .0005) and CRP (*p* = .0037)—highest in active disease, lowest in controls—but the active–inactive difference was non‐significant after false‐discovery‐rate correction.

**Conclusions:**

Perspiration CRP agreed moderately with serum CRP, and perspiration calprotectin with faecal calprotectin in ulcerative colitis only. Their high negative predictive values support the study as a non‐invasive adjunct to, not a substitute for, standard‐of‐care testing. All findings are preliminary, and the temporal‐dynamics results are hypothesis‐generating, needing confirmation in a larger, prospectively designed, endoscopy‐anchored cohort.

## HIGHLIGHTS

- A wearable sensor continuously measured perspiration CRP and calprotectin for up to 30 days in 52 patients with IBD.
- Perspiration CRP correlated moderately with serum CRP; perspiration calprotectin correlated with faecal calprotectin in ulcerative colitis only.
- Perspiration cutoffs showed high negative but modest positive predictive values.
- Ultradian biomarker dynamics are exploratory and require confirmation.

## Graphical abstract

A wearable perspiration sensor (IBD AWARE) worn on the forearm measured CRP and calprotectin (with TNFα and IL‐6 as exploratory analytes) approximately every 3 min in 52 patients with IBD over 3–30 days and in 9 non‐IBD controls over 19–32 days. Perspiration CRP showed moderate concordance with serum CRP, and perspiration calprotectin with faecal calprotectin in ulcerative colitis only; perspiration cutoffs had high negative but modest positive predictive values. In the ultradian‐dynamics panel, the vertical (*z*) axis denotes normalized ultradian‐band (4–12 h) spectral power; temporal‐dynamics findings are exploratory.

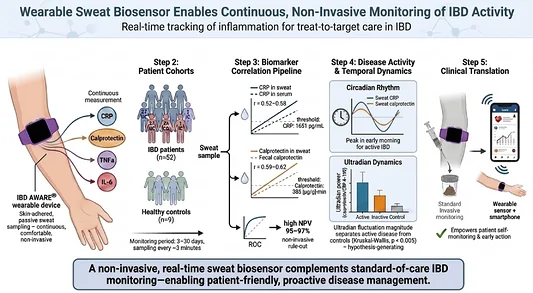

## 1 INTRODUCTION

Ulcerative colitis (UC) and Crohn's disease (CD), the two principal forms of inflammatory bowel disease (IBD), follow a chronic course of relapse and remission. An estimated 1 in 100 people in the United States are now affected by IBD,^1^ and its prevalence is increasing in both long‐industrialized and newly industrialized nations.^2^ Monitoring this growing population over many years calls for objective measures that can be obtained more often and more easily than current tests allow.

In routine care, bowel inflammation is reassessed at scheduled follow‐up visits and during symptomatic flares so that therapy can be adjusted toward defined treatment targets.^3^ The STRIDE‐II treat‐to‐target framework evaluates symptomatic, biochemical, and endoscopic targets in sequence^4^; how often and how conveniently inflammation can be measured objectively therefore determines how closely these targets can be tracked.

Each available assessment tool addresses a different aspect of disease activity. Colonoscopy, with or without histologic evaluation, remains the reference standard for mucosal inflammation; in CD, cross‐sectional imaging with MR enterography or intestinal ultrasound characterizes small‐bowel involvement and complications such as strictures and fistulas; and patient‐reported outcomes (PROs) record symptoms. Each tool also has a practical constraint: endoscopy is too resource‐intensive to repeat often, PROs are subjective and not specific for inflammation, and imaging requires trained personnel and captures a single time point. As a result, objective assessments are typically separated by long intervals.

Between endoscopic or imaging assessments, inflammation in IBD is most often tracked with serum CRP and faecal calprotectin,^5^, ^6^ which can be measured more frequently than colonoscopy allows. Both, however, depend on samples that patients may be reluctant to provide: blood collection is invasive and stool collection is inconvenient. In one study, only ∼50% of patients completed ordered faecal calprotectin tests, compared with ∼75% for blood tests,^7^ and a quality‐improvement study found that 33% of faecal calprotectin orders were never fulfilled, with non‐completion associated with no prior testing, distance to the laboratory, and discomfort with stool handling.^8^ Such barriers can lengthen the gaps between objective measurements of inflammation.

Wearable devices offer one way to collect objective data between clinic visits.^9^ Using wearable devices, Hirten et al.^9^ observed that heart rate and resting heart rate rose during inflammatory and symptomatic IBD flares, and wearable physiological sensing is under investigation across gastroenterology more broadly.^10^ Heart rate and similar physiological signals, however, are not specific to bowel inflammation and can change with many unrelated conditions. What is still lacking is a patient‐friendly way to track, on a continuous basis, biochemical markers that relate directly to IBD inflammatory activity.

Perspiration is a candidate biofluid for this purpose. In contrast to blood and stool, it reaches the skin surface passively and continuously, so it can be sampled without invasive collection, active sampling by the patient, or external laboratory processing. Several circulating inflammatory proteins can be detected in perspiration. Our group previously quantified calprotectin by ELISA in perspiration collected from six individuals (300–1300 ng/mL),^11^ and subsequent cross‐sectional and 2–4 day pilot studies with the IBD AWARE platform measured perspiration CRP, calprotectin, IL‐6, and TNFα in IBD outpatient and inpatient cohorts.^11^, ^12^, ^13^, ^14^ Those studies were limited to cross‐sectional sampling or a few days of wear.

A continuous signal can also be examined for temporal structure, both near‐24 h (circadian) patterns and faster, sub‐24 h (ultradian) fluctuations. Any such structure could arise from biological rhythms, immune‐cell trafficking, sleep‐wake behaviour, sweat physiology, or technical factors, and whether it carries clinical meaning for perspiration inflammatory biomarkers in IBD has not been established.

Building on those short‐term pilots, the present study used the IBD AWARE wearable (EnLiSense LLC, Allen, TX, USA) to monitor perspiration continuously for 3–30 days in patients with IBD and for 19–32 days in non‐IBD controls; some participants had also taken part in the earlier studies. Relative to that earlier work, this study combines multi‐week wear, a non‐IBD control group, paired serum CRP and faecal calprotectin comparator samples, and exploratory analyses of circadian and ultradian rhythms. The primary objective was to assess the feasibility of extended wear and the concordance of perspiration CRP and calprotectin with paired serum CRP and faecal calprotectin measurements. Secondary objectives were to estimate perspiration thresholds equivalent to conventional biochemical cutoffs and to characterize circadian patterns of the perspiration signal in biochemically active IBD, biochemically inactive IBD, and non‐IBD controls. As an exploratory objective, we examined whether ultradian features of the signal carried disease‐associated information. The study was not designed to establish clinical utility, predict relapse, guide or monitor therapy, assess adherence or treatment response, or replace standard‐of‐care testing, and the device was not validated against endoscopic or histologic disease activity.

## 2 METHODS

### 2.1 Study design and setting

This multicentre observational study enrolled participants at the University of Texas at Dallas, the Icahn School of Medicine at Mount Sinai, and the University of Chicago Medicine. Perspiration CRP and calprotectin were recorded continuously, with TNFα and IL‐6 recorded as exploratory analytes. Serum CRP and faecal calprotectin (FCAL) were obtained at the start and end of the study period. No publicly available datasets were used in this study.

### 2.2 Definitions of disease activity

Biochemical activity was defined by serum CRP: IBD patients with serum CRP > 5 mg/L were classified as biochemically active, and those whose serum CRP remained ≤5 mg/L throughout the study period as biochemically inactive. These labels describe biochemical activity only. Clinical activity, by contrast, refers to symptom‐based assessment (captured in this study by the clinical activity indices described below), whereas endoscopic and histological activity are separate dimensions of disease activity that were not assessed. Because serum CRP was used both to assign activity groups and to evaluate the CRP‐based wearable signal, the CRP results carry a degree of circularity, which is taken into account in their interpretation. Faecal calprotectin above 250 µg/g at any point during the study was considered elevated.^15^ Baseline clinical disease activity was scored at enrolment for every participant with a validated disease‐specific index—the Simple Clinical Colitis Activity Index (SCCAI) in UC and the Harvey–Bradshaw index (HBI) in CD (Table 1)—and these scores were used in the exploratory analyses relating perspiration dynamics to clinical (symptom‐based) disease activity.

**TABLE 1:** Baseline characteristics of study participants.

IBD (*n* = 52)		
**Disease subtype**		
Ulcerative colitis (UC)	27 (51.9%)	
Crohn's disease (CD)	24 (46.2%)	
Indeterminate colitis (IC)	1 (1.9%)	
Female	34 (65.4%)	
Male	18 (34.6%)	
Median age (years), range	33.5 (19–70)	
Median age of diagnosis (years), range	25 (10–65)	

Characteristic	UC (*n* = 27)	CD (*n* = 24)
**Disease activity index**	**SCCAI**	**HBI**
Remission	12 (0–2)	22 (<5)
Mild	10 (3–5)	2 (5–7)
Moderate	4 (6–11)	0 (8–16)
Severe	0 (>11)	0 (>16)
N/A	1	0
**Extent (Montreal classification)**		
N/A	3	0
L1	0	7
L2	0	8
L3	0	9
E1	7	–
E2	7	–
E3	10	–
**Complicated behaviour (Montreal)**	–	
B1	–	10
B2	–	10
B3	–	2
**Therapy class**		
Aminosalicylates	8	2
Corticosteroids	10	3
Immunomodulators	4	4
Biologics	15	15
Median baseline FCAL (µg/g), range	48.7 (6–5370)	47 (1.9–1635)
Median baseline serum CRP (mg/L), range	1.5 (.1–11.8)	1.7 (.1–19)
**Controls (*n* = 9)**		
Female	5	
Male	4	
Median age, years (range)	28 (18–51)	

*Note*: Values are *n* (%) or *n* unless otherwise indicated. Numbers in parentheses after activity categories give the index ranges defining each category. The participant with indeterminate colitis is included in the IBD totals but not in the subtype columns.

Abbreviation: B2, stricturing; B3, penetrating; behaviour B1, non‐stricturing, non‐penetrating; CRP, C‐reactive protein; E2, left‐sided colitis; E3, extensive colitis; FCAL, faecal calprotectin; HBI, Harvey–Bradshaw index; L2, colonic; L3, ileocolonic; location L1, ileal; Montreal extent E1, proctitis; N/A, not available; SCCAI, simple clinical colitis activity index.

### 2.3 Wearable perspiration sensor

Perspiration was sampled with IBD AWARE, a non‐invasive, proprietary wearable device worn on the antebrachial region of the arm throughout the study period. The sensor recorded a measurement approximately every 3 min, and data were uploaded to an external server whenever the wearer synced the device through the accompanying mobile application. A wicking patch at the skin interface continuously renews the sweat volume in contact with the affinity‐binding electrodes, so that only microlitre‐scale sweat volumes are needed for analyte detection, as validated previously.^11^, ^12^, ^13^, ^14^ Each ∼3 min reading is therefore an integrated biomarker signal over the preceding sampling interval, derived with a machine‐learning‐based signal processing method, rather than a single instantaneous concentration measurement.^12^, ^13^, ^14^ Sensors were worn continuously (24 h/day); each patch was worn for 6 days and replaced weekly over the full monitoring period. The IBD AWARE device is manufactured by EnLiSense LLC (Allen); additional technical information is available from the manufacturer (https://www.enlisense.com/resources). The device is intended for investigational use only.

### 2.4 Participants

Patients were enrolled under IRB 23–537 at the University of Texas at Dallas, IRBs 21–00428 & 23–00565 at Mount Sinai, and IRB 23–0863 at the University of Chicago. Each participant provided informed consent at the time and site of enrolment. Study data were not used to guide patient care.

The single participant with indeterminate colitis (IC; chronic colitis with overlapping features that could not be definitively categorized as UC or CD on clinical, endoscopic, radiological, and histological grounds) was included in the overall IBD counts and in the continuous‐monitoring and exploratory analyses, but not in the subtype‐specific activity‐index and Montreal classification summaries.

#### 2.4.1 Inclusion criteria

Eligible IBD participants were adults (≥18 years) able to give informed consent who had a confirmed diagnosis of CD, UC, or indeterminate colitis (IC). Eligible participants without IBD were adults (≥18 years) able to give informed consent who had never been diagnosed with CD, UC, or IC. All participants agreed to provide blood samples for the study and were able to use the IBD AWARE mobile app and devices.

#### 2.4.2 Exclusion criteria

Subjects excluded from this study were: known pregnant or currently lactating women, subjects with current malignancy or active treatment for previously diagnosed malignancy (except squamous and basal cell cancer of the skin), subjects with severe co‐morbidity (including immunodeficiency, recent myocardial infarction, or stroke, in the past 3 months), history of renal or hepatic failure, or sepsis. Non‐English‐speaking subjects were excluded, along with any subjects with a history of regular use of antidiarrheal agents, an active obstruction or a penetrating complication (i.e., abscess, phlegmon), and any active infection.

### 2.5 Statistical methods

Because two authors (S.P. and S.M.) hold a financial interest in the device manufacturer, the clinical reference labels (serum CRP, FCAL, and clinical activity indices) were fixed before the perspiration analyses, and the exploratory temporal analyses were evaluated by cross‐validation and label‐permutation testing rather than investigator judgement. The underlying data were verified by S.S., K.C.L., and S.P.; because S.P. holds a financial interest in the manufacturer, this internal verification does not constitute fully independent verification.

#### 2.5.1 Pairing of perspiration and reference measurements

For comparison with discrete serum measurements, perspiration CRP readings (recorded approximately every 3 min with the IBD AWARE device) were averaged over the 2 h following each serum collection, yielding 99 paired serum–perspiration CRP observations. For comparison with discrete FCAL measurements, perspiration calprotectin (SCAL) readings were summarized as the area under the curve (AUC) of perspiration concentration from 12 to 6 h before each stool collection, yielding 41 paired FCAL–SCAL observations. A window preceding stool collection was used to account for the accumulation of FCAL in the intestinal tract before stool passage; FCAL is also stable in stool for several days at room temperature. Of the windows tested (0–6 h, 0–8 h, 4–8 h, and 12–6 h before stool collection; Supporting Information S1), the 12–6 h window was selected empirically because it gave the strongest correlation with FCAL; because this selection was made in the same dataset, the resulting correlation estimates may be optimistic. Perspiration is predominantly aqueous (*ρ*≈1.0 g/mL), so perspiration calprotectin concentration was expressed as mass per unit mass (µg/g) to match the faecal calprotectin convention; the AUC integrated over the sampling window therefore has units of (µg/g)·min.

#### 2.5.2 Correlation, group comparison, threshold, and agreement analyses

Relationships between serum and perspiration CRP and TNFα (Supporting Information S2), and between FCAL and SCAL overall and within each disease subtype, were characterized with Pearson's (linear) and Spearman's (rank‐based, monotonic) correlation coefficients. The Mann–Whitney *U*‐test was used to compare perspiration CRP between paired observations with normal (<5 mg/L) and elevated (>5 mg/L) serum CRP, and perspiration calprotectin AUC between paired observations with controlled (<250 µg/g) and elevated (>250 µg/g) FCAL. Optimal perspiration CRP and calprotectin cutoffs corresponding to the serum CRP (5 mg/L) and FCAL (250 µg/g) thresholds, respectively, were identified by receiver operating characteristic (ROC) analysis, and sensitivity, specificity, positive predictive value (PPV), and negative predictive value (NPV) were computed at each cutoff.

Agreement between perspiration and reference measurements was evaluated with Bland–Altman analysis, from which the bias (mean difference) and 95% limits of agreement (bias ± 1.96 SD) are reported. For CRP, perspiration values (pg/mL) were placed on the serum scale (mg/L) by dividing by a factor of approximately 300, the ratio of the perspiration CRP cutoff (1551 pg/mL) to the serum CRP threshold (5 mg/L). Perspiration calprotectin AUC ((µg/g)·min) and FCAL (µg/g) are expressed on different scales, so the calprotectin Bland–Altman analysis is descriptive only. In the exploratory clinical‐activity analysis, the UC and CD correlation coefficients were compared using Fisher's r‐to‐z transformation. Two‐sided tests were used throughout, with statistical significance set at *p* < .05. Analyses were carried out in GraphPad Prism and Python.

### 2.6 Differential rhythmic patterns using CircaCompare

Between‐group differences in the rhythmic behaviour of perspiration inflammatory biomarker levels were assessed with CircaCompare, a chronobiology method developed by Parsons et al.^16^ For two datasets collected over 24 h, CircaCompare estimates and statistically tests differences in three rhythm parameters: the MESOR (midline‐estimating statistic of rhythm), the rhythm‐adjusted mean level of biomarker expression over 24 h; the amplitude, the distance from the MESOR to the peak of the fitted rhythm; and the acrophase, the time at which that peak occurs.

Differential rhythmicity was represented as the 15 min aggregate mean within each 24 h period, that is, as a single circadian cycle, averaged over the days the IBD AWARE device was worn by each subject and across all subjects. Patients were stratified by biochemical activity as defined above.

### 2.7 Exploratory ultradian rhythm and multianalyte temporal‐feature classification analysis

An exploratory analysis tested whether the faster, sub‐24‐h dynamics of the perspiration biomarker stream contained disease‐relevant information. It included the whole monitored cohort: 52 IBD subjects grouped by biochemical activity (15 active and 37 inactive, using the serum CRP cutoff of 5 mg/L defined above) and 9 controls without IBD. For each subject and analyte (CRP, calprotectin, TNFα, IL‐6), a Lomb–Scargle periodogram was computed on the continuous perspiration time series to estimate normalized spectral power across periods of 2 to 48 h. Ultradian structure was summarized per subject by the normalized power integrated over the ultradian band (4–12 h period) and by a panel of complementary cosinor and spectral descriptors—dominant ultradian period, peak power, the ultradian fraction of total subcircadian‐plus‐circadian power, cosinor amplitude and relative amplitude, acrophase, percent rhythm (cosinor *R* ^2^), and MESOR—with the corresponding circadian‐band (20–28 h) descriptors computed for reference. Metrics were compared across the three groups (active, inactive, control) using the Kruskal–Wallis test, and pairwise group contrasts (active vs. inactive, active vs. control, inactive vs. control) were quantified with Hedges' g and Cliff's delta effect sizes with bootstrap 95% confidence intervals and Mann–Whitney U *p*‐values; *p*‐values were corrected across comparisons using the Benjamini–Hochberg false discovery rate (reported as q). Subject‐level bootstrap resampling was used so that repeated 3 min readings were not treated as independent observations; it does not add independent patients, and the effective sample comprised 15 biochemically active, 37 inactive, and 9 control participants. Supporting Information S6 presents the pairwise Hedges’ g effect sizes and their 95% confidence intervals as a forest plot.

A temporal classifier of biochemical activity (active vs. inactive) was then constructed from the ultradian‐power features. Because the earlier per‐marker temporal feature set (spectral power, volatility, drift) was prone to overfitting at this sample size, the feature representation was restricted to the log‐transformed ultradian‐band power of each analyte, optionally combined with the corresponding time‐averaged (static) level. An L2‐regularized logistic‐regression classifier with balanced class weights was trained for each analyte individually and for the four‐analyte panel jointly, and evaluated by leave‐one‐out cross‐validation (LOO‐CV), reporting the area under the receiver operating characteristic curve (AUC) with bootstrap 95% confidence intervals. Each ultradian‐power classifier was compared against a classifier built on static, time‐averaged marker levels using the same cross‐validation procedure and clinical labels, and statistical significance relative to chance was assessed by label‐permutation testing with the full LOO pipeline re‐fit on each permutation.

To relate the perspiration ultradian signal to clinical (symptomatic) disease activity, a single composite sweat ultradian‐dynamics score was computed for each subject as the mean of the standardized (z‐scored) log ultradian‐band power across the four analytes, so that the four markers contributed on a common scale. This composite score was correlated separately within each disease subtype against the disease‐specific baseline clinical activity index—the SCCAI in UC and the HBI in CD—using the Spearman rank correlation coefficient, with the correlation strength, two‐sided *p*‐value, and number of subjects with both an analyzable recording and a recorded clinical score reported for each subtype. This analysis was exploratory and hypothesis‐generating rather than the primary, prespecified analysis of the study, and the modest sample size and the imbalance between Active and Inactive groups limit its interpretation.

## 3 RESULTS

### 3.1 Study cohort and feasibility

Perspiration CRP and calprotectin were recorded continuously for 3–30 days in 52 subjects diagnosed with ulcerative colitis (UC; *n* = 27), Crohn's disease (CD; *n* = 24), or indeterminate colitis (IC; *n* = 1) (Table 1). Forty‐seven of the 52 enrolled IBD subjects (90.4%) completed the full monitoring protocol, showing that continuous multi‐week wear was feasible in this cohort. In a subset of inpatients (*n* = 6), serum CRP was measured daily; in the remaining inpatients (*n* = 14) and in the outpatient subgroup (n = 32), serum CRP and FCAL were measured at the start and end of the study period. The control cohort comprised 9 subjects without IBD, whose perspiration CRP and calprotectin were recorded for 19–32 days (Table 1) and whose serum CRP was measured at the start and end of monitoring. Because serum CRP remained within the normal range in the large majority of controls, no separate correlation analysis was performed in this subgroup. Baseline therapy classes were recorded only to describe the cohort (Table 1); 13 of the 52 IBD participants (25%) were receiving corticosteroids at baseline.

### 3.2 Serum CRP and skin perspiration CRP correlation and threshold

In all IBD patients combined, serum and skin perspiration CRP were moderately correlated (Pearson's *r* = .58; Spearman's *r* = .52; 99 paired observations; Figure 1a).

**FIGURE 1:**
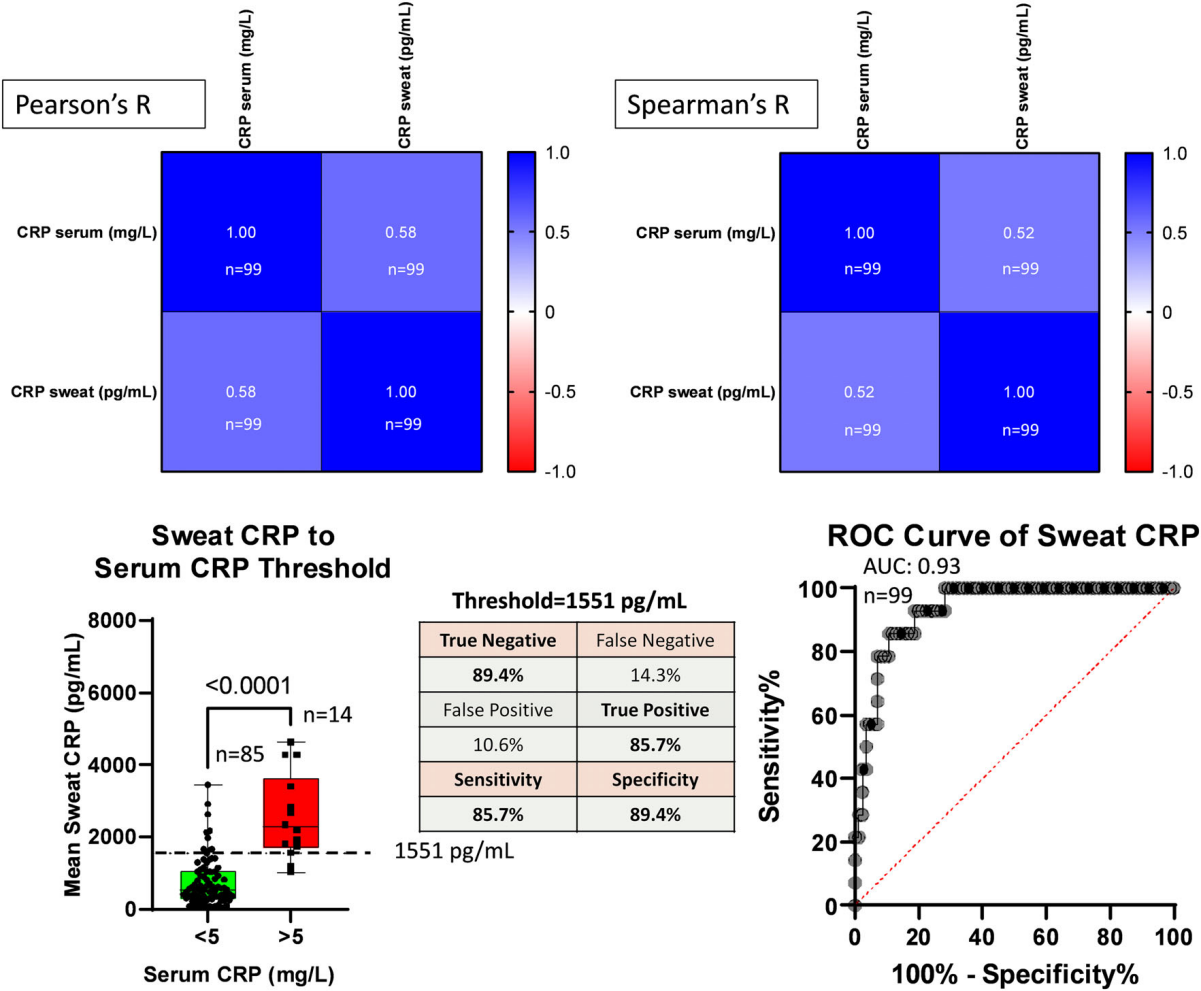
Concordance between serum and perspiration CRP. (a) Serum CRP and skin perspiration CRP correlation: correlation matrices (Pearson's, left; Spearman's, right) for serum CRP (mg/L) and perspiration CRP (pg/mL; 2 h average after serum collection) in all IBD participants (*n* = 99 paired observations). (b) Serum CRP and skin perspiration CRP threshold: perspiration CRP in observations with serum CRP <5 mg/L (*n* = 85) and >5 mg/L (*n* = 14) (box plots with individual values; Mann–Whitney test; dashed line, 1551 pg/mL cutoff), classification table at the cutoff, and ROC curve (AUC  .93); the red dotted diagonal indicates chance performance.

A skin perspiration CRP of 1551 pg/mL corresponded to the serum CRP threshold of 5 mg/L (Figure 1b); this cutoff was identified from a receiver operating characteristic (ROC) curve (AUC  .93) built from serum CRP measurements in patients wearing the IBD AWARE device. At this cutoff, perspiration CRP identified elevated serum CRP with 85.7% sensitivity and 89.4% specificity in the IBD patient cohort, and perspiration CRP was higher in observations with serum CRP > 5 mg/L than in those with serum CRP < 5 mg/L (Mann–Whitney *p* < .0001). The positive predictive value (PPV) for elevated serum CRP was 57%, and the negative predictive value (NPV) for normal serum CRP was 97%.

### 3.3 Faecal calprotectin and skin perspiration calprotectin correlation and threshold

In UC patients, faecal calprotectin (FCAL) and skin perspiration calprotectin (SCAL) were moderately correlated (Pearson's *r* = .62; Spearman's *r* = .59; Figure 2a). No comparable correlation was found in CD patients (Supporting Information S1).

**FIGURE 2:**
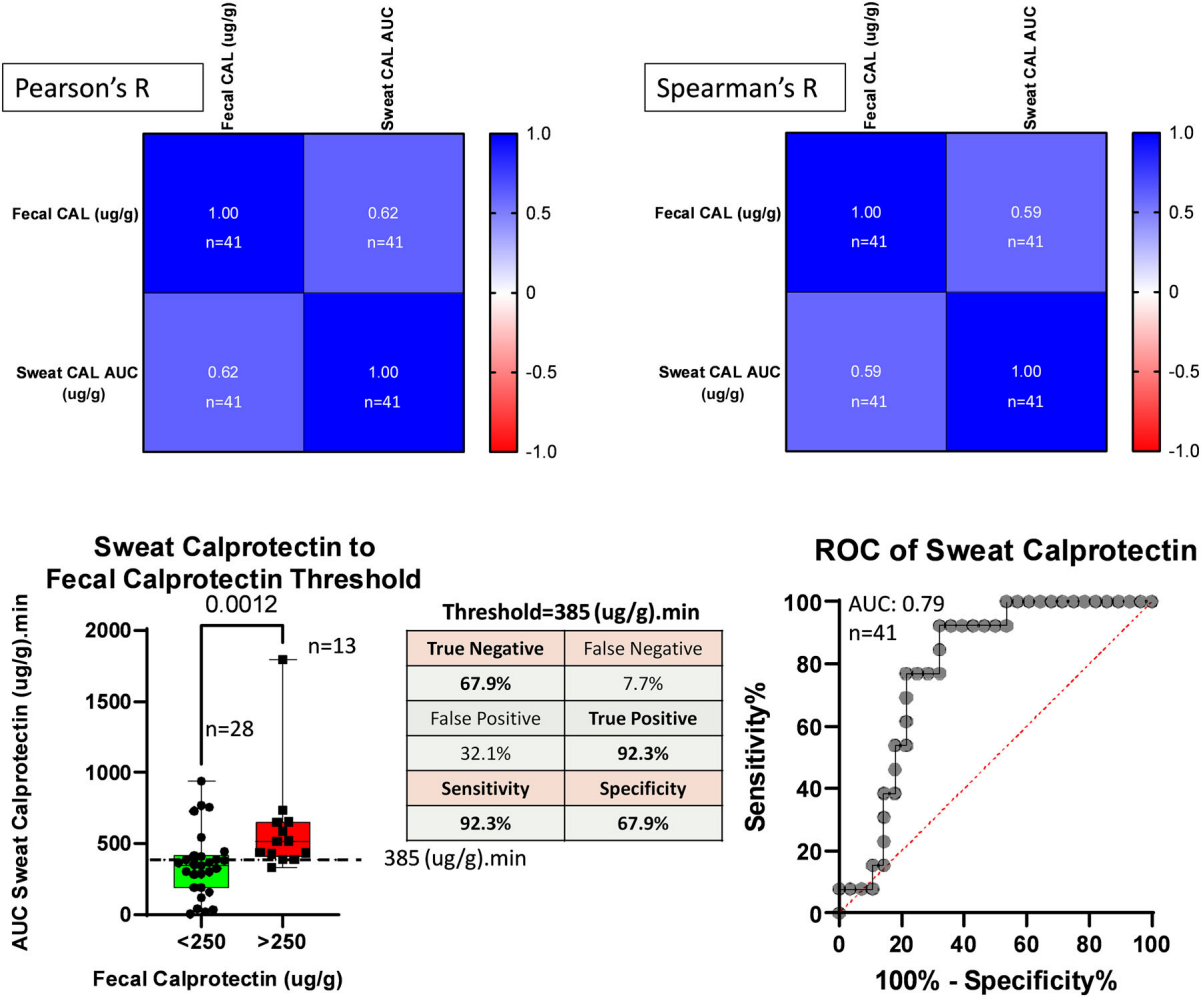
Concordance between faecal and perspiration calprotectin. (a) Faecal calprotectin and skin perspiration calprotectin correlation: correlation matrices (Pearson's, left; Spearman's, right) for FCAL (µg/g) and perspiration calprotectin AUC ((µg/g)·min, 12‐to‐6 h before stool collection; *n* = 41 paired observations). (b) Faecal calprotectin and skin perspiration calprotectin threshold: perspiration calprotectin AUC in observations with FCAL < 250 µg/g (*n* = 28) and >250 µg/g (*n* = 13) (box plots with individual values; Mann–Whitney test; dashed line, 385 (µg/g)·min cutoff), classification table at the cutoff, and ROC curve (AUC  .79); the red dotted diagonal indicates chance performance.

An optimal SCAL cutoff of 385 (µg/g)·min corresponded to the FCAL threshold of 250 µg/g (Figure 2b); this cutoff was derived from a receiver operating characteristic (ROC) curve (AUC  .79) relating FCAL measured in patients wearing the IBD AWARE device to SCAL aggregated over the 12‐to‐6 h window before stool sample collection. At this cutoff, elevated FCAL was identified with 92.3% sensitivity and 67.9% specificity in the IBD patient cohort, and perspiration calprotectin AUC was higher in observations with FCAL > 250 µg/g than in those with FCAL < 250 µg/g (Mann–Whitney *p* = .0012). The PPV for elevated FCAL was 57%, and the NPV for controlled calprotectin was 95%.

### 3.4 Agreement between perspiration and conventional biomarkers

To characterize agreement beyond correlation, Bland–Altman analysis was performed on the paired measurements (Figure 3). For CRP, the mean difference between serum and perspiration measurements was small (bias −.72 mg/L), but the 95% limits of agreement (−6.07 to +4.63 mg/L; *n* = 99) were wide relative to the 5 mg/L clinical threshold. For calprotectin, there was a small positive bias (79.22 (µg/g)·min) with 95% limits of agreement of −1850 to +2008 (*n* = 41); because the two calprotectin measures are on different scales, this comparison is descriptive. In both cases, the spread widened at higher concentrations, consistent with the recognized biological and analytical variability of CRP and faecal calprotectin.

**FIGURE 3:**
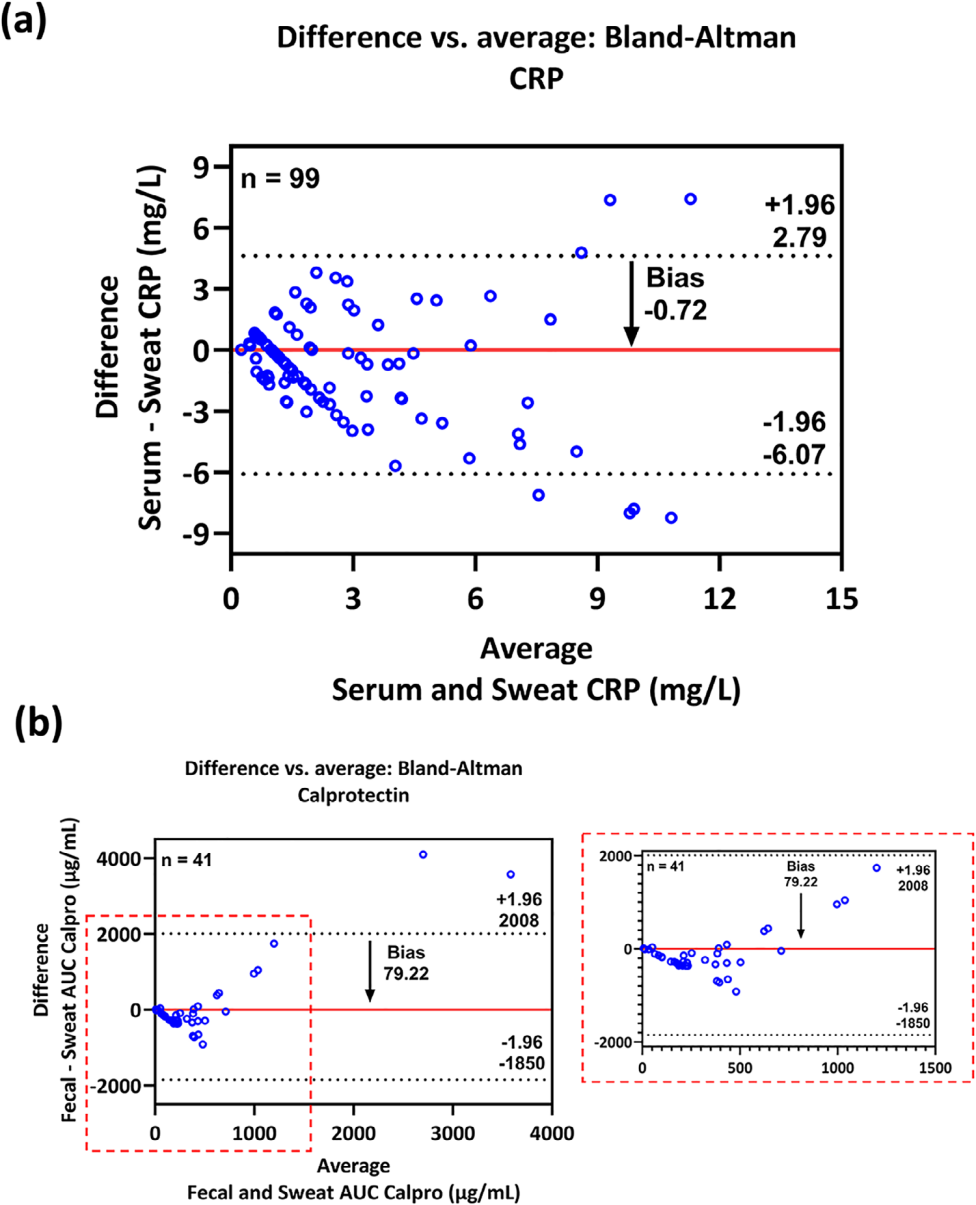
Bland–Altman agreement analysis between paired perspiration and conventional biomarker measurements. Difference‐versus‐average plots for (a) serum versus perspiration CRP (*n* = 99; perspiration CRP converted to mg/L by dividing by ∼300) and (b) faecal versus perspiration calprotectin (*n* = 41). Solid red line, mean difference (bias); dotted lines, 95% limits of agreement (bias ± 1.96 SD); the boxed inset in (b) magnifies the clinically relevant lower concentration range. Bias was −.72 mg/L for CRP (limits of agreement −6.07 to +4.63 mg/L) and 79.22 (µg/g)·min for calprotectin (limits of agreement −1850 to +2008); the calprotectin comparison is descriptive because the two measures are on different scales.

### 3.5 Differential rhythmicity of skin perspiration CRP by biochemical disease activity

Differential rhythmicity of skin perspiration CRP is shown for IBD patients with elevated biochemical activity as defined by their serum CRP (Active), IBD patients with biochemically inactive disease (Inactive), and control subjects without IBD (non‐IBD) in Figure 4. Active IBD showed higher skin perspiration CRP than both Inactive IBD and non‐IBD subjects. The acrophase of peak CRP expression in Active and Inactive IBD patients occurred in the morning, at approximately 6:45 AM and 7:15 AM, respectively, whereas non‐IBD participants showed a single peak with an acrophase at approximately 2:45 PM. Active IBD patients also had the highest amplitude of 340.181 pg/mL with a MESOR of 1243.651 pg/mL, compared with an amplitude of 147.113 pg/mL (*p* = .0001) and a MESOR of 950.521 pg/mL (*p* = .0001) for Inactive IBD and with an amplitude of 302.642 pg/mL (*p* = .0288) and a MESOR of 1143.583 pg/mL (*p* = .0001) for non‐IBD (Supporting Information S3). The CRP MESOR ordering (active > non‐IBD > inactive) is not a monotonic activity gradient, which limits interpretation of MESOR as a direct activity marker; treatment exposure and sweat availability are potential unmodelled contributors. In Supporting Information S3, pairwise differences are reported as first group minus second group.

**FIGURE 4:**
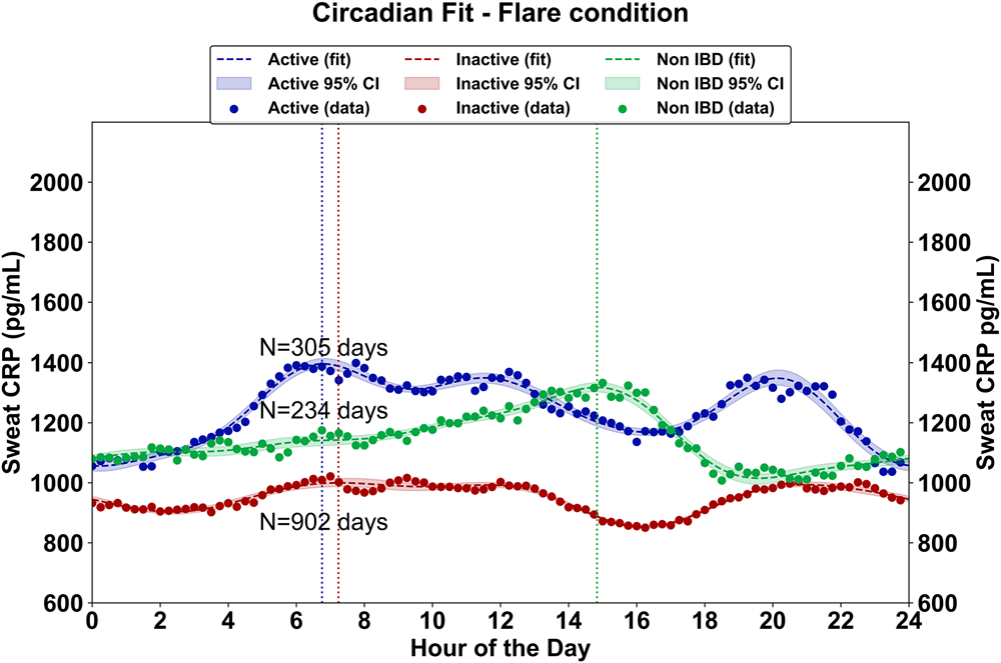
Differential rhythmicity of skin perspiration CRP by biochemical disease activity. Points show 15 min aggregate means over a single 24 h cycle for Active (serum CRP > 5 mg/L), Inactive, and non‐IBD participants; dashed lines show fitted rhythms with 95% confidence bands; vertical dotted lines mark the acrophase of each group. *N* represents the aggregated monitoring days across active, inactive, and non‐IBD subjects.

### 3.6 Differential rhythmicity of skin perspiration calprotectin by biochemical disease activity

Differential rhythmicity of skin perspiration calprotectin is shown for Active, Inactive, and Non‐IBD participants (Figure 5). Patients were stratified according to serum CRP rather than FCAL, because FCAL reflects stool accumulated over hours to days and cannot be aligned with a 24 h cycle.

**FIGURE 5:**
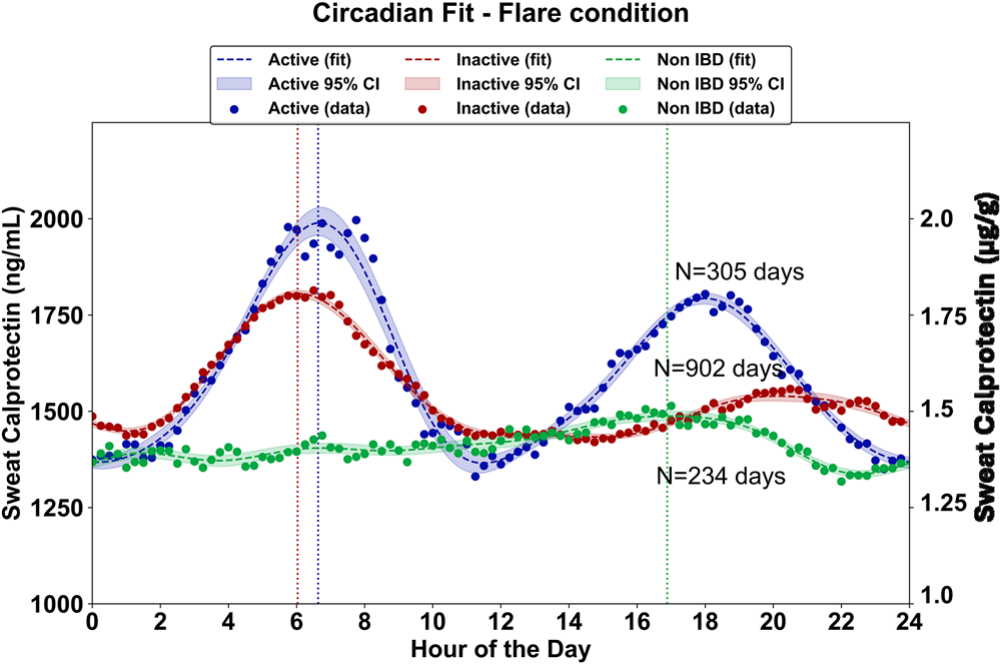
Differential rhythmicity of skin perspiration calprotectin by biochemical disease activity. Points show 15 min aggregate means over a single 24 h cycle for active (serum CRP > 5 mg/L), inactive, and non‐IBD participants; dashed lines show fitted rhythms with 95% confidence bands; vertical dotted lines mark the acrophase of each group; *N*, aggregated monitoring days per group. Left axis, ng/mL; right axis, equivalent µg/g.

Active IBD showed higher perspiration calprotectin than Inactive IBD and non‐IBD, and Inactive IBD showed higher levels than non‐IBD. The acrophase of peak calprotectin expression in Active and Inactive IBD occurred between 6:00 and 6:30 AM, similar to perspiration CRP. In contrast to perspiration CRP, an additional peak occurred in both active and inactive IBD in the evening (approximately 6:00–8:00 PM) and was more pronounced in Active IBD. Non‐IBD participants showed a comparatively flat profile over 24 h. Active IBD patients also had the highest amplitude of 624.140 ng/mL with a MESOR of 1598.23 ng/mL, compared with an amplitude of 372.361 ng/mL (*p* = .0001) and a MESOR of 1541.641 ng/mL (*p* = .0001) for Inactive IBD and with an amplitude of 150.271 ng/mL (*p* = .0001) and a MESOR of 1411.622 ng/mL (*p* = .0001) for non‐IBD (Supporting Information S4).

### 3.7 Exploratory ultradian rhythm dynamics and multianalyte classification of disease activity

Beyond the approximately 24 h circadian pattern, the continuous perspiration stream was examined for faster, sub‐24 h (ultradian) structure across the three groups (52 IBD subjects: 15 active and 37 inactive by serum CRP; 9 controls without IBD). Normalized ultradian‐band (4–12 h) spectral power differed significantly across the three groups for perspiration calprotectin (Kruskal–Wallis *p* = .0005) and CRP (*p* = .0037), and was ordered active > inactive > control for both markers (Figure 6); the differences were not significant for TNFα (*p* = .29) or IL‐6 (*p* = .14). The separation was driven predominantly by the contrast between IBD and controls: for calprotectin, ultradian power was markedly higher in active than control participants (Hedges’ *g* = 1.39, 95% CI  .94–3.20; *q* = .008) and in inactive than control (*g* = .94,  .60–1.58; *q* = .011), with a smaller, nonsignificant active‐versus‐inactive difference (*g* = .38, *q* = .11). CRP followed the same pattern (active vs. control *g* = 1.02, *q* = .011; inactive vs. control *g* = .75, *q* = .035; active vs. inactive *g* = .37, *q* = .11); the full set of pairwise Hedges' *g* effect sizes with 95% confidence intervals across all four analytes is shown in Supporting Information S6. Several complementary ultradian descriptors reinforced the calprotectin finding: the ultradian peak power (*p* = .0003), cosinor amplitude (*p* = .0029), relative amplitude (*p* = .0006), percent rhythm (cosinor *R* ^2^, *p* = .0004), and the ultradian fraction of total power (*p* = .023) all differed across groups and were highest in active disease, whereas MESOR, dominant period, and acrophase did not differ (Supporting Information S5). By contrast, the group MESOR (mean level) of calprotectin did not separate the groups (*p* = .54), indicating that the ultradian‐band differences reflect the temporal structure of the signal rather than its average level. These circadian and ultradian findings are framed as exploratory temporal structure: a periodogram identifies periodicity but cannot determine whether that structure is biological or technical, because unmeasured factors such as sweat rate, hydration, temperature, and activity can generate 4–12 h fluctuations.

**FIGURE 6:**
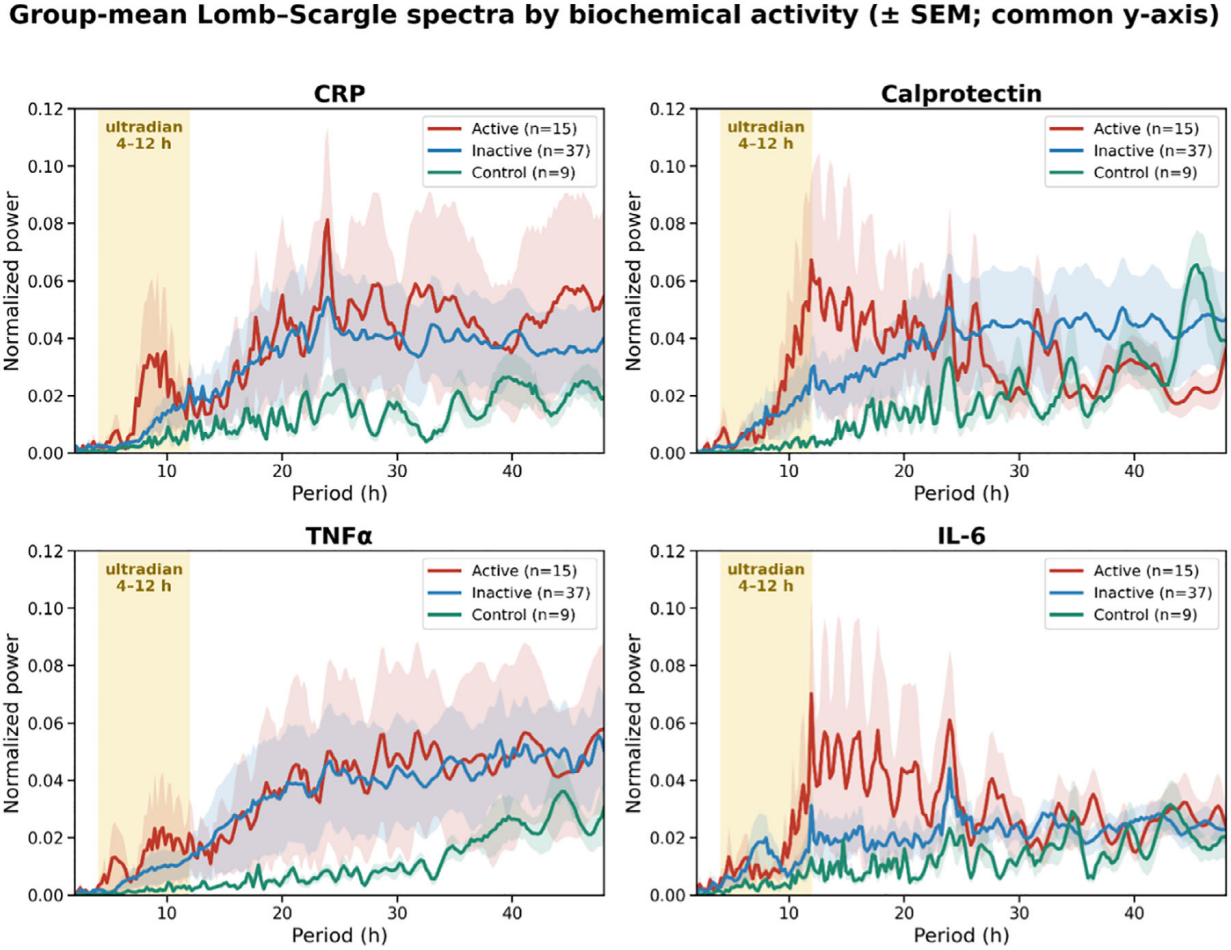
Group‐mean ultradian rhythm structure of perspiration biomarkers by biochemical disease activity. Group‐mean Lomb–Scargle periodograms (±standard error of the mean) for skin perspiration CRP, calprotectin, TNFα, and IL‐6 in biochemically active (*n* = 15) and Inactive (*n* = 37) IBD and in controls without IBD (*n* = 9), across oscillation periods of 2–48 h; the ultradian band (4–12 h) is shaded. Normalized ultradian‐band power is highest in Active disease and lowest in controls, most prominently for perspiration calprotectin and CRP (three‐group Kruskal–Wallis *p* = .0005 and *p* = .0037, respectively). Per‐metric group statistics are provided in Supporting Information S5, and the pairwise Hedges' g effect sizes (95% CI) are shown in Supporting Information S6. This analysis is exploratory.

To test whether this ultradian structure carried classifiable information about biochemical activity, a leave‐one‐out cross‐validated classifier was built from the log ultradian‐band power of the analytes (active vs. inactive; *n* = 52). Perspiration calprotectin was the strongest single feature, discriminating active from inactive disease with an AUC of  .66 (95% CI  .49–.83; label‐permutation *p* = .058), followed by the four‐analyte ultradian‐power panel (AUC  .64, 95% CI  .48–.79; *p* = .073) and CRP (AUC  .63, 95% CI:  .43–.81; *p* = .086). Combining CRP and calprotectin ultradian power did not improve on calprotectin alone (AUC  .59, 95% CI:  .39–.78), and TNFα and IL‐6 ultradian power did not discriminate activity (AUC < .5). In direct comparison, a classifier built on static, time‐averaged levels of the same four analytes performed near chance (AUC  .53, 95% CI:  .34–.70; *p* = .375); the numerical difference of approximately  .13 AUC relative to the calprotectin ultradian‐power model was not formally tested (Figure 7). No classifier performed significantly better than chance (all permutation *p* > .05), all confidence intervals included  .5, and the biochemically active group was small (15 of 52); these results therefore do not establish that the device can classify disease activity.

**FIGURE 7:**
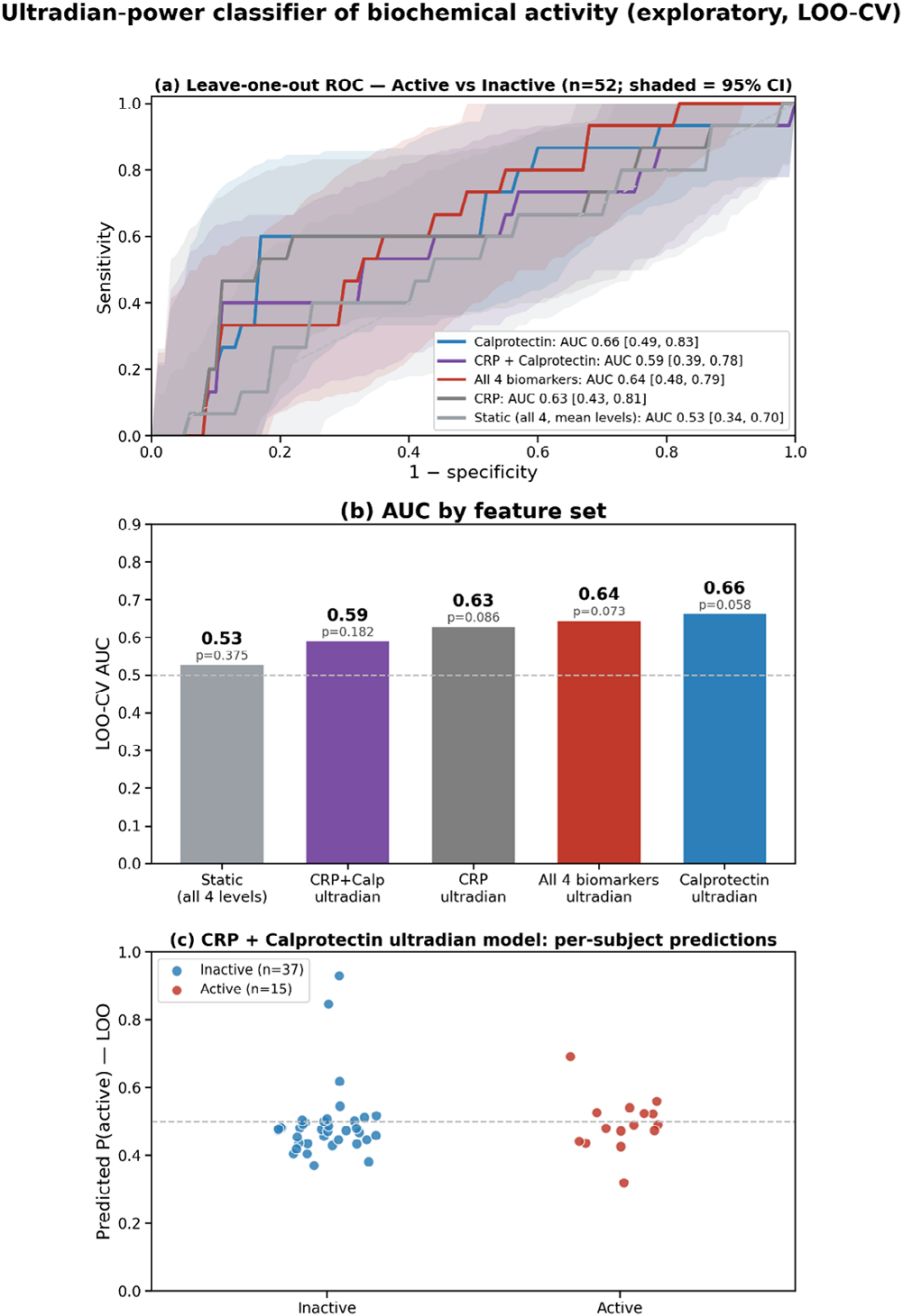
Exploratory ultradian‐power classification of biochemical disease activity (active vs. inactive IBD; *n* = 52; 15 active, 37 inactive). Leave‐one‐out cross‐validated classifiers built from the log ultradian‐band (4–12 h) power of the perspiration analytes. (a) Receiver operating characteristic curves with shaded 95% bootstrap confidence bands, each labelled with its area under the curve (AUC) and 95% CI, for the calprotectin ultradian‐power classifier (AUC  .66, 95% CI:  .49–.83), the CRP‐plus‐calprotectin two‐marker ultradian‐power classifier (.59,  .39–.78), the all‐four‐biomarker (CRP+Calp+IL6+TNFα) ultradian‐power panel (.64,  .48–.79), the CRP ultradian‐power classifier (.63,  .43–.81), and a static all‐four‐biomarker classifier using time‐averaged marker levels (.53,  .34–.70). (b) Leave‐one‐out AUC by feature set, with the label‐permutation *p*‐value shown above each bar: calprotectin ultradian power is the strongest single feature (AUC  .66; *p* = .058), followed by the all‐four‐biomarker panel (.64; *p* = .073), CRP (.63; *p* = .086), the CRP‐plus‐calprotectin combination (.59; *p* = .182), and the static all‐four‐biomarker classifier (.53; *p* = .375). No feature set reached *p* < .05, consistent with the exploratory, underpowered nature of the activity‐classification analysis; the CRP‐plus‐calprotectin combination did not improve on calprotectin alone. (c) Per‐subject leave‐one‐out predicted probability of active disease from the CRP‐plus‐calprotectin ultradian‐power model, by biochemical activity group. Results are exploratory and hypothesis‐generating.

### 3.8 Exploratory association between perspiration ultradian dynamics and clinical activity indices

The composite sweat ultradian‐dynamics score correlated with the baseline SCCAI in UC (Spearman *r* = +.55, *p* = .004, *n* = 25) but not with the baseline HBI in CD (Spearman *r* = +.09, *p* = .70, *n* = 23) (Figure 8). The two correlation coefficients did not differ significantly (Fisher r‐to‐z, *z* = 1.73, *p* = .083). Both indices are symptom‐based, and each correlation rests on a single baseline score per participant; this analysis is exploratory.

**FIGURE 8:**
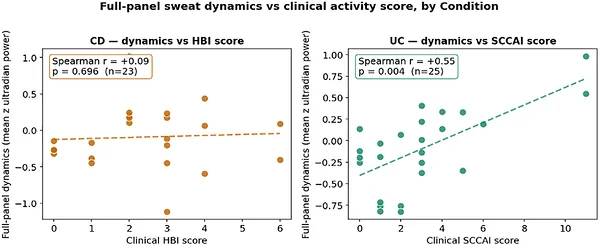
Exploratory association of perspiration ultradian dynamics with clinical activity indices. A composite sweat ultradian‐dynamics score (mean of standardized log ultradian‐band [4–12 h] power across CRP, calprotectin, TNFα, and IL‐6) is plotted against the baseline clinical activity index for each participant. Left, Crohn's disease: Harvey–Bradshaw index (HBI; Spearman *r* = +.09, *p* = .70, *n* = 23). Right, ulcerative colitis: simple clinical colitis activity index (SCCAI; Spearman *r* = +.55, *p* = .004, *n* = 25). Dashed lines, least‐squares fits shown for visualization only. This analysis is exploratory.

## 4 DISCUSSION

In this extended feasibility and biomarker‐concordance study, continuous perspiration sensing over multi‐day to multi‐week periods was feasible in most participants, with 47 of 52 completing the protocol. Perspiration CRP agreed moderately with serum CRP (*r* = .52–.58), and perspiration calprotectin agreed moderately with faecal calprotectin in UC (*r* = .59–.62) but not in CD. The perspiration cutoffs equivalent to serum CRP 5 mg/L and FCAL 250 µg/g had high NPVs (97% and 95%) and modest PPVs (57%). Circadian and ultradian analyses detected temporal structure in the perspiration signal that differed between groups, chiefly between IBD and non‐IBD participants. Below, we separate what these data directly show from interpretations that remain hypotheses.

### 4.1 Concordance with standard‐of‐care biomarkers

These moderate correlations need to be read alongside the imperfect performance of the reference biomarkers themselves, neither of which tracks objective measures of disease activity exactly.^17^ Serum CRP correlates only moderately with endoscopic activity (*r*≈.42–.48),^5^ consistent with its known limitations as a surrogate for mucosal inflammation,^18^ whereas faecal calprotectin tracks endoscopy more closely^19^ but performs poorly in isolated small‐bowel CD.^20^, ^21^ Our question was therefore whether perspiration measurements follow the direction of conventional biomarkers, not whether the two matrices yield analytically identical values. These data indicate moderate concordance without interchangeability: for CRP, the Bland–Altman limits of agreement were wide relative to the 5 mg/L threshold, and because few observations within each subtype had elevated serum CRP, the CRP analysis could be performed only in the combined IBD cohort.

Serum CRP > 5 mg/L has been associated with active inflammation in IBD^22^ and with relapse after withdrawal of anti‐TNF therapy,^23^ and CRP concentration is related to endoscopic and histologic activity.^24^ In our cohort, the high NPV and modest PPV of the corresponding perspiration cutoffs mean that positive perspiration results were often false positives, whereas negative results generally matched a normal reference value. Predictive values depend on prevalence and on how the cohort was sampled, and they may be lower in populations with more frequent flares; moreover, the cutoffs were derived and tested in the same dataset without external validation, so their performance is probably overestimated. A negative perspiration result does not rule out intestinal inflammation, confirm clinical remission, or indicate freedom from relapse risk, and these data do not show that blood or stool testing can be safely deferred on the basis of a normal wearable result. At this stage, perspiration CRP and calprotectin are best viewed as candidate non‐invasive screening signals for further evaluation, not as substitutes for serum CRP or faecal calprotectin.

### 4.2 Differences between ulcerative colitis and Crohn's disease

Perspiration calprotectin correlated with FCAL in UC but not in CD. A likely contributor is the location‐dependent performance of FCAL itself, which predicts endoscopic activity in UC but not in small‐bowel CD,^20^ correlates with endoscopic activity chiefly in colonic and ileocolonic CD,^25^ and remains low in isolated ileal disease even when that disease is severe.^21^ Our CD cohort comprised 7 participants with isolated ileal (L1), 8 with colonic (L2), and 9 with ileocolonic (L3) disease, so FCAL was probably a less informative reference standard in CD than in UC. We did not investigate the biological basis of the association seen in UC. Because CRP is synthesized mainly by hepatocytes in response to systemic cytokine signalling, and calprotectin is released by activated neutrophils and monocytes and is not gut‐specific, perspiration levels of both analytes most plausibly reflect systemic rather than gut‐specific inflammatory activity. The paired FCAL–SCAL dataset (*n* = 41) was too small for CRP‐adjusted or CRP‐stratified analyses, so the UC association may be partly explained by both measures increasing with overall systemic inflammation. Proposed mechanisms, such as transfer from dermal interstitial fluid or local release by myeloid cells in the skin, remain hypotheses that will need dedicated studies adjusting for serum CRP, disease location, medication exposure, and endoscopic activity.

### 4.3 Exploratory temporal dynamics

Ultradian (4–12 h) spectral power of perspiration calprotectin and CRP differed across groups in the order active > inactive > control. This separation, however, came mainly from the contrast between IBD and controls: after false‐discovery‐rate correction, the active‐versus‐inactive differences were not significant, and no ultradian‐power classifier distinguished active from inactive disease significantly better than chance. Similarly, the composite ultradian score correlated with the SCCAI in UC but not with the HBI in CD, yet the two subtypes did not differ significantly, and both indices are symptom‐based scores recorded once at baseline that do not directly measure mucosal inflammation. Regarding interpretation, a periodogram detects periodicity but cannot distinguish biological from technical sources: sweat rate, hydration, temperature, physical activity, sleep–wake behaviour, and device–skin interface factors can each produce fluctuations on these timescales, and none was modelled here. Ultradian structure is clinically relevant in other physiological systems, for example, the hypothalamic–pituitary–adrenal axis,^26^ and ultradian features derived from wearables vary with disease state in other conditions.^27^ Whether perspiration inflammatory biomarkers carry comparable information in IBD is a hypothesis that requires testing in a larger, prospectively designed cohort in which these confounders are measured.

This study did not examine whether continuous perspiration biomarker monitoring could detect biochemical change earlier, enable more timely clinical intervention, or lessen the burden of blood and stool testing,^7^, ^8^ because treatment changes, relapse, hospitalization, and adherence outcomes were not evaluated. Any clinical role would first require prospective validation in a larger cohort that is more diverse in disease activity and demographics, links the perspiration signal directly to endoscopic activity and relapse, and tests whether device output can inform clinical decision‐making.

### 4.4 Strengths and limitations

Strengths of this study include continuous, multi‐analyte perspiration monitoring over weeks rather than days, extending earlier short‐term IBD AWARE studies^12^, ^13^, ^14^; a non‐IBD control group; comparison of perspiration measurements against paired serum and faecal reference biomarkers; and the use of cross‐validation and label‐permutation testing in the exploratory classification analyses. Perspiration volume was adequate for analysis, in line with prior validation of microlitre‐scale sweat sufficiency for this platform.^11^, ^12^, ^13^, ^14^

The study also has important limitations, grouped here by source. Design and cohort: the cohort was modest in size, predominantly young (median age 33.5 years for IBD participants and 28 years for controls), and weighted toward patients in remission. The activity groups were small and imbalanced (15 biochemically active and 37 inactive IBD participants, and 9 controls), which reduces statistical power, precludes adequately powered subtype‐ and location‐specific analyses, and restricts generalizability. Because UC and CD are distinct diseases with different patterns of inflammation, the combined biochemically active group, defined by serum CRP alone, should not be taken to represent a uniform inflammatory burden across IBD. Reference standards and sampling: no concurrent endoscopic or histologic reference standard was available, and activity was defined either biochemically (serum CRP) or by symptom‐based indices (SCCAI/HBI), each an imperfect surrogate for mucosal inflammation, particularly in CD; using serum CRP both to define activity groups and as the comparator for perspiration CRP also introduces circularity. Reference biomarkers were sampled sparsely (at baseline and study end for most participants, and daily in six inpatients), whereas perspiration was sampled approximately every 3 min, so agreement could be assessed only at coinciding timepoints, and it cannot be determined whether fluctuations between reference collections reflect true inflammatory change or artefact. Statistical dependence and data‐driven selection: some participants contributed more than one paired observation to the correlation, ROC, and Bland–Altman analyses, and the circadian comparisons pooled monitoring days across participants; because within‐participant clustering was not accounted for, the *p*‐values may be overstated. The perspiration cutoffs and the 12‐to‐6 h calprotectin aggregation window were also selected and evaluated in the same dataset, without external validation. Confounders: physiological and technical factors, including physical activity, ambient temperature, hydration, sweat rate, skin physiology, and device–skin interface variability, were neither measured nor modelled. Medication exposure was recorded descriptively only; 13 of the 52 IBD participants were receiving corticosteroids at baseline, which can suppress serum CRP and faecal calprotectin and may influence perspiration biomarkers, and dose, timing, tapering, and treatment response were not modelled. Exploratory analyses: the ultradian, classifier, and clinical‐activity analyses were exploratory and involved multiple comparisons, and their key active‐versus‐inactive contrasts did not survive correction. Conflicts of interest: two authors hold a financial interest in the device manufacturer; although reference labels were fixed before analysis and the exploratory analyses used cross‐validation and permutation testing, the data were not verified by a fully independent team. These limitations should be addressed in a larger, prospectively designed, demographically and clinically diverse cohort with concurrent endoscopic assessment, denser reference sampling, prespecified medication covariates, and measurement of physiological confounders.

## 5 CONCLUSIONS

In this feasibility pilot, patients with IBD wore a perspiration sensor continuously for up to several weeks. Measurements from the IBD AWARE device were moderately concordant with serum CRP and, in ulcerative colitis but not Crohn's disease, with faecal calprotectin. Perspiration thresholds equivalent to standard‐of‐care biochemical cutoffs were estimated and showed high negative but modest positive predictive values; these cutoffs require external validation. Temporal structure identified by circadian and ultradian analyses differed mainly between IBD and non‐IBD participants; these exploratory associations may reflect physiological and technical factors as well as disease, are hypothesis‐generating only, and do not represent validated clinical performance. On this basis, perspiration biomarker sensing warrants further evaluation as a possible non‐invasive adjunct to standard‐of‐care monitoring, not a replacement for it. Treatment adjustment, medication adherence, treatment response, relapse prediction, hospitalization, and other outcome‐linked endpoints were not evaluated in this study. Prospective studies should test whether wearable perspiration biomarker monitoring provides clinically actionable information when combined with detailed treatment exposure data, adherence measures, standard‐of‐care biomarkers, and endoscopic or histologic outcomes.

## AUTHOR CONTRIBUTIONS

S.P. and S.M. developed the study concept. S.S., K.C.L., A.R.M., R.P.H., S.M., B.E.S., D.T.R., and S.P. assisted with the drafting of the manuscript. S.S., K.C.L., A.R., A.R.M., Z.D.F., R.P.H., J.W., D.H., S.M., B.E.S., D.T.R., and S.P. critically revised the manuscript for important intellectual content. S.S., K.C.L., A.R., A.R.M., Z.D.F., R.P.H., J.W., D.H., S.M., B.E.S., D.T.R., and S.P. provided final approval of the version of the manuscript to be published and agreed to be accountable for all aspects of the work. All authors approve of the authorship list. All authors had full access to all the data in the manuscript and had final responsibility for the decision to submit for publication. All authors have approved the final draft of the manuscript. S.S., K.C.L. and S.P. have verified the underlying data. The authors report no writing assistance with the manuscript.

## CONFLICT OF INTEREST STATEMENT

S.P. and S.M. have a significant interest in EnLiSense LLC, a company that may have a commercial interest in the results of this research and technology. The potential individual conflict of interest has been reviewed and managed by the University of Texas at Dallas and played no role in the study design, the collection, analysis, and interpretation of data, the writing of the report, or the decision to submit the report for publication. D.T.R. has received grant support from Takeda and served as a consultant for AbbVie, Abgenomics, Arena Pharmaceuticals, Bellatrix Pharmaceuticals, Boehringer Ingelheim Ltd, Bristol‐Myers Squibb, Celgene, Syneos, Dizal Pharmaceuticals, Genentech/Roche, Gilead Sciences, Ichnos Sciences S.A., InDex Pharmaceuticals, Iterative Scopes, Janssen Pharmaceuticals, Lilly, Pfizer, Prometheus Laboratories, Reistone, Takeda, and Techlab Inc. RPH has served on advisory boards for Bristol Myers Squibb and reports consulting fees from Bristol Myers Squibb and Abbvie, stock options from Salvo Health, and research support from Janssen, Intralytix, Enlisense, and the Crohn's and Colitis Foundation. BES has received consulting fees from Abbvie, Alimentiv, Adiso Therapeutics, Agomab, Amgen, AnaptysBio, Astra Zeneca, Biolojic Design, Biora Therapeutics, Boehringer Ingelheim, Celltrion, Equillium, Ensho Therapeutics, Enthera, Enveda Biosciences, Evommune, Ferring, Fzata, Galapagos, Genentech (Roche), Gilead Sciences, GlaxoSmithKline, Gossamer Bio, Imhotex, Immunyx Therapeutics, Index Pharmaceuticals, Innovation Pharmaceuticals, Kaleido, Kallyope, Merck & Co., Microba, Microbiotica, Mirador Therapeutics, Mitsubishi Tanabe Pharma, Mobius Care, Morphic Therapeutics, MRM Health, Nexus Therapeutics, Nimbus Discovery, Odyssey Therapeutics, Palisade Bio, Prometheus Biosciences, Prometheus Laboratories, Protagonist Therapeutics, Q32 Bio, Rasayana Therapeutics, Recludix Therapeutics, Reistone Biotherapeutics, Sanofi, Sorriso Pharmaceuticals, Surrozen, Target RWE, Teva, TLL Pharmaceutical, TR1X, and Union Therapeutics; consulting and speaking fees from Abivax; consulting and speaking fees and other support from Lilly; research grants, consulting and speaking fees and other support from Bristol Myers Squibb, Janssen/J & J Innovative Medicine, Pfizer, and Takeda; research grants and consulting fees from Theravance Biopharma; and consulting fees, stock and stock options from Ventyx Biopharma. The remaining authors declare no conflict of interest.

## ETHICS STATEMENT AND CONSENT TO PARTICIPATE

This study was approved by the Institutional Review Board at the University of Texas at Dallas (IRB 23–537), the Icahn School of Medicine at Mount Sinai (IRBs 21–00428 and 23–00565), and the University of Chicago (IRB 23–0863). All participants gave informed consent at the time and site of enrolment. Study data were not used to guide patient care.

## CONSENT FOR PUBLICATION

No individually identifiable participant data, images, or case details are presented in this manuscript.

## Supporting information



Supporting Information

## Data Availability

The de‐identified continuous perspiration biomarker data and the corresponding serum/faecal biomarker measurements underlying this study can be requested from the corresponding author; reasonable requests will be considered subject to institutional and ethical approval, because the raw dataset contains detailed longitudinal biometric information from a limited‐size clinical cohort. No publicly available datasets were used in this study.
