# Supplementary material for: A wearable perspiration biosensor for continuous biomarker monitoring in inflammatory bowel disease: Feasibility, concordance, and exploratory temporal dynamics
Source: Clin Transl Med. 2026 Nov 14;16(11):e70896. doi: 10.1002/ctm2.70896 (PMC13770216; doi:10.1002/ctm2.70896)
Supplement: Supplementary file 1 [file CTM2-16-e70896-s001.docx]

**A Wearable Perspiration Biosensor to Support Treat-to-Target Monitoring in Inflammatory Bowel Disease**

Annapoorna Ramasubramanya^1^, MS, Kai-Chun Lin^1^, PhD, Sarah Shahub^1^, PhD, Alexandra R. McDermott^2^, BS, Zachary D. Fine^2^, BA, Robert P. Hirten^3^, MD, Jessica Whang^3^, MS, Drew Helmus^3^, PhD, Sriram Muthukumar^4^, PhD, Bruce E. Sands^3^, MD, David T. Rubin^2^, MD & Shalini Prasad^1,4^, PhD

^1^Bioengineering, University of Texas at Dallas, 800 West Campbell Rd., Richardson, TX, USA

^2^The University of Chicago Medicine Inflammatory Bowel Disease Center, Chicago, IL, USA

^3^The Dr. Henry D. Janowitz Division of Gastroenterology, Icahn School of Medicine at Mount Sinai, New York, NY, USA

^4^EnLiSense LLC, Allen, TX, USA

*Corresponding Author / Guarantor of the Article*

Dr. Shalini Prasad. Email: Shalini.Prasad@utdallas.edu; address: 860 N Loop Rd BSB11.102C, Richardson, TX, 75080; phone: +1 (972) 883-4247

**Online Resources**

*Online Resource 1*

Determination of the optimal temporal window for correlation with accumulated FCAL based on empirical analysis of the patients' AUC of SCAL using different times ranging from 0 to 6 hours, 0 to 8 hours, 4 to 8 hours, and 12 to 6 hours.

*Online Resource 2*

Serum TNFα and skin perspiration TNFα correlation and threshold.

*Online Resource 3*

MESOR and amplitude estimates of perspiration CRP by disease status.

| Metric | Value | p-value (subject-level bootstrap) | Significant (p<0.05, bootstrap) |
| --- | --- | --- | --- |
| Active MESOR | 1243.651 pg/mL | — | — |
| Inactive MESOR | 950.521 pg/mL | — | — |
| Non-IBD MESOR | 1143.583 pg/mL | — | — |
| MESOR: Active vs Inactive | 293.09 pg/mL | 0.0001 | Yes |
| MESOR: Active vs Non-IBD | -100.013 pg/mL | 0.0001 | Yes |
| MESOR: Inactive vs Non-IBD | 193.077 pg/mL | 0.0001 | Yes |
| Amplitude: Active | 340.181 pg/mL | — | — |
| Amplitude: Inactive | 147.113 pg/mL | — | — |
| Amplitude: Non-IBD | 302.642 pg/mL | — | — |
| Amplitude: Active vs Inactive | 191.887 pg/mL | 0.0001 | Yes |
| Amplitude: Active vs Non-IBD | -37.485 pg/mL | 0.0288 | Yes |
| Amplitude: Inactive vs Non-IBD | 154.402 pg/mL | 0.0001 | Yes |

*Online Resource 4*

MESOR and amplitude estimates of perspiration calprotectin by disease status.

| Metric | Value | p-value (subject-level bootstrap) | Significant (p<0.05, bootstrap) |
| --- | --- | --- | --- |
| Active MESOR | 1598.23 ng/mL | — | — |
| Inactive MESOR | 1541.641 ng/mL | — | — |
| Non-IBD MESOR | 1411.622 ng/mL | — | — |
| MESOR: Active vs Inactive | 56.602 ng/mL | 0.0001 | Yes |
| MESOR: Active vs Non-IBD | -186.587 ng/mL | 0.0001 | Yes |
| MESOR: Inactive vs Non-IBD | -129.985 ng/mL | 0.0001 | Yes |
| Amplitude: Active | 624.140 ng/mL | — | — |
| Amplitude: Inactive | 372.361 ng/mL | — | — |
| Amplitude: Non-IBD | 150.271 ng/mL | — | — |
| Amplitude: Active vs Inactive | 256.002 ng/mL | 0.0001 | Yes |
| Amplitude: Active vs Non-IBD | -477.985 ng/mL | 0.0001 | Yes |
| Amplitude: Inactive vs Non-IBD | -221.983 ng/mL | 0.0001 | Yes |

Reported p-values are derived from subject-level bootstrap resampling (resampling subjects, not individual time points), which respects the non-independence of repeated within-subject measurements. Pooled time-point t-tests on the raw measurement stream are not reported, as treating autocorrelated within-subject time points as independent observations would constitute pseudoreplication and yield spuriously extreme p-values.

*Online Resource 5*

Exploratory three-group ultradian analysis and ultradian-power classifier performance across the monitored cohort (52 IBD subjects: 15 Active, 37 Inactive by the serum CRP cutoff of 5 mg/L; 9 controls without IBD). All results are exploratory. Group values are medians with interquartile range. Three-group comparisons use the Kruskal-Wallis test; pairwise effect sizes are Hedges’ g and Cliff’s delta with bootstrap 95% confidence intervals and Mann-Whitney U p-values, corrected across comparisons by the Benjamini-Hochberg false discovery rate (q). Classifier AUCs are leave-one-out cross-validated with bootstrap 95% confidence intervals; permutation p-values re-fit the full leave-one-out pipeline on permuted labels.

Table O5.1. Pairwise ultradian-band (4-12 h) power effect sizes.

| Analyte | Comparison | g (95% CI) | Cliff δ | p | q (BH-FDR) |
| --- | --- | --- | --- | --- | --- |
| CRP | Active vs Inactive | 0.37 (-0.22-1.08) | 0.32 | 0.075 | 0.113 |
| CRP | Active vs Control | 1.02 (0.66-2.31) | 0.75 | 0.003 | 0.011 |
| CRP | Inactive vs Control | 0.75 (0.36-1.39) | 0.55 | 0.012 | 0.035 |
| Calprotectin | Active vs Inactive | 0.38 (-0.17-1.11) | 0.36 | 0.045 | 0.109 |
| Calprotectin | Active vs Control | 1.39 (0.94-3.20) | 0.85 | 0.001 | 0.008 |
| Calprotectin | Inactive vs Control | 0.94 (0.60-1.58) | 0.68 | 0.002 | 0.011 |
| TNFα | Active vs Inactive | 0.14 (-0.40-0.80) | 0.11 | 0.531 | 0.580 |
| TNFα | Active vs Control | 0.60 (0.18-1.28) | 0.38 | 0.136 | 0.181 |
| TNFα | Inactive vs Control | 0.45 (0.09-0.81) | 0.27 | 0.213 | 0.255 |
| IL-6 | Active vs Inactive | 0.08 (-0.51-0.77) | 0.06 | 0.747 | 0.747 |
| IL-6 | Active vs Control | 0.65 (0.11-1.31) | 0.45 | 0.074 | 0.113 |
| IL-6 | Inactive vs Control | 0.60 (0.24-1.11) | 0.39 | 0.072 | 0.113 |

Table O5.2. Ultradian and circadian rhythm metrics by group (medians, IQR).

| Marker | Metric | Unit | Active (n=15) | Inactive (n=37) | Control (n=9) | KW p |
| --- | --- | --- | --- | --- | --- | --- |
| CRP | Ultradian period | h | 11.17 (9.77–11.54) | 11.17 (10.48–11.72) | 11.26 (10.07–11.81) | 0.9423 |
| CRP | Ultradian power (norm.) |  | 0.0050 (0.0030–0.0055) | 0.0031 (0.0026–0.0043) | 0.0020 (0.0016–0.0026) | 0.0027 |
| CRP | Ultradian peak power |  | 0.027 (0.021–0.049) | 0.024 (0.017–0.039) | 0.024 (0.012–0.033) | 0.2332 |
| CRP | Ultradian fraction U/(U+C) |  | 0.218 (0.163–0.247) | 0.169 (0.117–0.215) | 0.143 (0.129–0.181) | 0.0630 |
| CRP | Ultradian amplitude | pg/mL | 151 (117–189) | 145 (112–203) | 142 (134–187) | 0.8851 |
| CRP | Ultradian relative amplitude | % | 14.0 (10.8–23.0) | 15.7 (11.5–25.9) | 17.0 (10.2–19.7) | 0.9467 |
| CRP | Ultradian acrophase (into cycle) | h | 5.70 (3.47–7.90) | 6.03 (2.57–8.38) | 5.09 (3.39–5.57) | 0.5471 |
| CRP | % rhythm (cosinor R²) | % | 2.1 (1.0–3.6) | 1.9 (0.9–3.2) | 2.2 (0.9–2.8) | 0.8409 |
| CRP | MESOR (mean level) | pg/mL | 898 (732–1423) | 853 (688–1132) | 817 (682–1340) | 0.7293 |
| CRP | Circadian period | h | 24.78 (23.91–27.38) | 23.63 (21.30–24.59) | 25.38 (21.64–25.58) | 0.1076 |
| CRP | Circadian power (norm.) |  | 0.0155 (0.0119–0.0291) | 0.0178 (0.0131–0.0241) | 0.0120 (0.0107–0.0143) | 0.1055 |
| CRP | Circadian relative amplitude | % | 21.9 (12.8–29.6) | 19.7 (11.5–29.4) | 13.4 (9.5–18.9) | 0.2570 |
| CRP | Recording span | h | 793 (691–1252) | 704 (545–1056) | 643 (626–696) | 0.2817 |
| Calprotectin | Ultradian period | h | 11.35 (10.24–11.72) | 10.91 (10.48–11.54) | 10.74 (10.57–11.17) | 0.5483 |
| Calprotectin | Ultradian power (norm.) |  | 0.0048 (0.0029–0.0061) | 0.0032 (0.0021–0.0043) | 0.0014 (0.0012–0.0015) | 0.0005 |
| Calprotectin | Ultradian peak power |  | 0.050 (0.023–0.065) | 0.025 (0.017–0.036) | 0.010 (0.008–0.011) | 0.0003 |
| Calprotectin | Ultradian fraction U/(U+C) |  | 0.205 (0.121–0.393) | 0.192 (0.135–0.238) | 0.111 (0.097–0.120) | 0.0231 |
| Calprotectin | Ultradian amplitude | ng/mL | 256 (199–358) | 182 (153–257) | 128 (92.3–153) | 0.0029 |
| Calprotectin | Ultradian relative amplitude | % | 17.0 (13.9–23.2) | 12.2 (10.7–17.2) | 8.8 (8.1–11.0) | 0.0006 |
| Calprotectin | Ultradian acrophase (into cycle) | h | 6.74 (2.76–7.84) | 6.10 (4.02–8.62) | 6.12 (3.19–6.80) | 0.5912 |
| Calprotectin | % rhythm (cosinor R²) | % | 4.2 (2.2–6.2) | 2.2 (1.4–4.1) | 1.0 (0.8–1.2) | 0.0004 |
| Calprotectin | MESOR (mean level) | ng/mL | 1510 (1396–1626) | 1432 (1310–1672) | 1435 (1094–1571) | 0.5367 |
| Calprotectin | Circadian period | h | 23.63 (21.81–25.20) | 23.63 (21.64–25.79) | 23.44 (21.82–24.20) | 0.8934 |
| Calprotectin | Circadian power (norm.) |  | 0.0138 (0.0076–0.0269) | 0.0120 (0.0083–0.0235) | 0.0101 (0.0092–0.0120) | 0.5511 |
| Calprotectin | Circadian relative amplitude | % | 15.1 (7.9–25.9) | 11.8 (7.6–16.3) | 11.3 (8.0–15.0) | 0.6386 |
| Calprotectin | Recording span | h | 793 (691–1252) | 704 (545–1056) | 643 (626–696) | 0.2817 |
| TNFα | Ultradian period | h | 10.74 (9.68–11.30) | 10.74 (10.07–11.44) | 10.32 (10.07–11.09) | 0.5046 |
| TNFα | Ultradian power (norm.) |  | 0.0018 (0.0015–0.0029) | 0.0016 (0.0012–0.0028) | 0.0017 (0.0011–0.0019) | 0.3675 |
| TNFα | Ultradian peak power |  | 0.019 (0.009–0.026) | 0.014 (0.008–0.020) | 0.010 (0.008–0.011) | 0.1645 |
| TNFα | Ultradian fraction U/(U+C) |  | 0.158 (0.128–0.208) | 0.124 (0.093–0.201) | 0.159 (0.143–0.174) | 0.1914 |
| TNFα | Ultradian amplitude | pg/mL | 0.3 (0.2–0.4) | 0.3 (0.3–0.4) | 0.3 (0.3–0.3) | 0.9872 |
| TNFα | Ultradian relative amplitude | % | 6.2 (4.0–9.7) | 5.1 (4.2–7.1) | 6.0 (5.3–6.9) | 0.7209 |
| TNFα | Ultradian acrophase (into cycle) | h | 6.41 (3.95–7.88) | 4.58 (2.39–7.59) | 8.14 (5.42–9.35) | 0.0958 |
| TNFα | % rhythm (cosinor R²) | % | 1.6 (0.9–2.2) | 1.1 (0.7–2.0) | 0.8 (0.7–1.2) | 0.1862 |
| TNFα | MESOR (mean level) | pg/mL | 5.2 (4.4–5.7) | 5.7 (5.0–6.5) | 5.4 (5.3–5.6) | 0.2870 |
| TNFα | Circadian period | h | 24.20 (22.90–25.38) | 24.98 (23.44–27.27) | 26.41 (24.00–27.49) | 0.3497 |
| TNFα | Circadian power (norm.) |  | 0.0123 (0.0104–0.0158) | 0.0123 (0.0082–0.0154) | 0.0072 (0.0041–0.0094) | 0.0171 |
| TNFα | Circadian relative amplitude | % | 5.9 (4.2–7.9) | 5.7 (3.8–7.8) | 5.8 (4.5–6.9) | 0.9218 |
| TNFα | Recording span | h | 793 (691–1252) | 704 (545–1056) | 643 (626–696) | 0.2817 |
| IL-6 | Ultradian period | h | 11.63 (8.32–11.91) | 10.16 (8.26–11.91) | 11.00 (8.66–11.17) | 0.6993 |
| IL-6 | Ultradian power (norm.) |  | 0.0052 (0.0036–0.0069) | 0.0045 (0.0035–0.0070) | 0.0039 (0.0024–0.0046) | 0.1586 |
| IL-6 | Ultradian peak power |  | 0.036 (0.022–0.056) | 0.036 (0.026–0.048) | 0.024 (0.014–0.027) | 0.0572 |
| IL-6 | Ultradian fraction U/(U+C) |  | 0.217 (0.175–0.262) | 0.221 (0.159–0.281) | 0.244 (0.167–0.326) | 0.8693 |
| IL-6 | Ultradian amplitude | pg/mL | 0.5 (0.4–0.6) | 0.5 (0.4–0.7) | 0.3 (0.3–0.5) | 0.0308 |
| IL-6 | Ultradian relative amplitude | % | 8.2 (6.0–9.3) | 8.5 (6.7–10.7) | 5.1 (4.6–7.8) | 0.0269 |
| IL-6 | Ultradian acrophase (into cycle) | h | 8.21 (2.85–9.94) | 5.03 (2.29–7.38) | 6.52 (3.08–7.90) | 0.2538 |
| IL-6 | % rhythm (cosinor R²) | % | 2.9 (2.4–4.8) | 3.5 (2.1–4.5) | 2.2 (1.2–2.7) | 0.0590 |
| IL-6 | MESOR (mean level) | pg/mL | 6.2 (5.7–6.8) | 6.4 (5.9–6.8) | 5.9 (5.8–6.3) | 0.3306 |
| IL-6 | Circadian period | h | 24.00 (21.56–24.69) | 24.20 (23.44–25.79) | 24.20 (23.63–24.78) | 0.6710 |
| IL-6 | Circadian power (norm.) |  | 0.0198 (0.0099–0.0236) | 0.0182 (0.0128–0.0247) | 0.0097 (0.0074–0.0161) | 0.0684 |
| IL-6 | Circadian relative amplitude | % | 6.8 (2.1–12.0) | 7.3 (4.8–11.3) | 4.7 (3.6–9.5) | 0.5401 |
| IL-6 | Recording span | h | 793 (691–1252) | 704 (545–1056) | 643 (626–696) | 0.2817 |

Table O5.3. Ultradian-power versus static-level classifier performance (Active vs Inactive; n=52).

| Classifier feature set | LOO-CV AUC (95% CI) | Permutation p |
| --- | --- | --- |
| Calprotectin – ultradian power | 0.66 (0.48-0.84) | 0.058 |
| Four-analyte panel – ultradian power | 0.64 (0.48-0.80) | 0.073 |
| CRP – ultradian power | 0.63 | — |
| TNFα – ultradian power | 0.43 | — |
| IL-6 – ultradian power | 0.39 | — |
| CRP – static (time-averaged) level | 0.59 | — |
| Calprotectin – static level | 0.31 | — |
| Four-analyte panel – static levels | 0.53 | — |

Note: the ultradian-power classifier improves on the static full-panel classifier (AUC 0.53), with perspiration calprotectin ultradian power the strongest single feature (AUC 0.66; permutation p=0.058). Effects are modest and borderline in significance given the imbalanced sample (15 Active vs 37 Inactive) and should be interpreted as hypothesis-generating. The Active-versus-Inactive ultradian-power differences did not survive Benjamini-Hochberg correction; the IBD-versus-control differences did.

*Online Resource 6*

Pairwise effect sizes (Hedges’ g with 95% confidence intervals) for the normalized ultradian-band (4-12 hour) power of each perspiration analyte, comparing Active versus Inactive IBD, Active IBD versus controls, and Inactive IBD versus controls. Positive values indicate greater ultradian power in the first-named group. Asterisks denote uncorrected Mann-Whitney p<0.05; Benjamini-Hochberg false-discovery-rate-corrected q-values are reported in Online Resource 5. Effect sizes were largest and most consistently significant for perspiration calprotectin and CRP, driven predominantly by the contrast between IBD and controls.

**Online Resource 6. Ultradian-power pairwise effect sizes (Hedges’ g, 95% CI) by analyte and group contrast.** This analysis is exploratory.
